# Prognostic value of a novel artificial intelligence-based coronary computed tomography angiography-derived ischaemia algorithm for patients with suspected coronary artery disease

**DOI:** 10.1093/ehjci/jead339

**Published:** 2023-12-12

**Authors:** Sarah Bär, Takeru Nabeta, Teemu Maaniitty, Antti Saraste, Jeroen J Bax, James P Earls, James K Min, Juhani Knuuti

**Affiliations:** Turku PET Centre, Turku University Hospital, University of Turku, Kiinamyllynkatu 4-8, 20520 Turku, Finland; Department of Cardiology, Bern University Hospital Inselspital, Bern, Switzerland; Department of Cardiology, Leiden University Medical Center, Leiden, The Netherlands; Turku PET Centre, Turku University Hospital, University of Turku, Kiinamyllynkatu 4-8, 20520 Turku, Finland; Department of Clinical Physiology, Nuclear Medicine, and PET, Turku University Hospital, Hämeentie 11, 20540 Turku, Finland; Turku PET Centre, Turku University Hospital, University of Turku, Kiinamyllynkatu 4-8, 20520 Turku, Finland; Heart Center, Turku University Hospital, University of Turku, Turku, Finland; Department of Cardiology, Leiden University Medical Center, Leiden, The Netherlands; Cleerly Inc., NewYork, NY, USA; Cleerly Inc., NewYork, NY, USA; Turku PET Centre, Turku University Hospital, University of Turku, Kiinamyllynkatu 4-8, 20520 Turku, Finland; Department of Clinical Physiology, Nuclear Medicine, and PET, Turku University Hospital, Hämeentie 11, 20540 Turku, Finland

**Keywords:** artificial intelligence, coronary computed tomography angiography, non-invasive imaging, ischaemia, prognosis

## Abstract

**Aims:**

Coronary computed tomography angiography (CTA) imaging is used to diagnose patients with suspected coronary artery disease (CAD). A novel artificial intelligence-guided quantitative computed tomography ischaemia algorithm (AI-QCT_ischaemia_) aims to identify myocardial ischaemia directly from CTA images and may be helpful to improve risk stratification. The aims were to investigate (i) the prognostic value of AI-QCT_ischaemia_ amongst symptomatic patients with suspected CAD entering diagnostic imaging with coronary CTA and (ii) the prognostic value of AI-QCT_ischaemia_ separately amongst patients with no/non-obstructive CAD (≤50% visual diameter stenosis) and obstructive CAD (>50% visual diameter stenosis).

**Methods and results:**

For this cohort study, AI-QCT_ischaemia_ was calculated by blinded analysts amongst patients with suspected CAD undergoing coronary CTA. The primary endpoint was the composite of death, myocardial infarction (MI), or unstable angina pectoris (uAP) (median follow-up 6.9 years). A total of 1880/2271 (83%) patients had conclusive AI-QCT_ischaemia_ result. Patients with an abnormal AI-QCT_ischaemia_ result (*n* = 509/1880) vs. patients with a normal AI-QCT_ischaemia_ result (*n* = 1371/1880) had significantly higher crude and adjusted rates of the primary endpoint [adjusted hazard ratio (HR_adj_) 1.96, 95% confidence interval (CI) 1.46–2.63, *P* < 0.001; covariates: age/sex/hypertension/diabetes/smoking/typical angina]. An abnormal AI-QCT_ischaemia_ result was associated with significantly higher crude and adjusted rates of the primary endpoint amongst patients with no/non-obstructive CAD (*n* = 1373/1847) (HR_adj_ 1.81, 95% CI 1.09–3.00, *P* = 0.022), but not amongst those with obstructive CAD (*n* = 474/1847) (HR_adj_ 1.26, 95% CI 0.75–2.12, *P* = 0.386) (*P*-interaction = 0.032).

**Conclusion:**

Amongst patients with suspected CAD, an abnormal AI-QCT_ischaemia_ result was associated with a two-fold increased adjusted rate of long-term death, MI, or uAP. AI-QCT_ischaemia_ may be useful to improve risk stratification, especially amongst patients with no/non-obstructive CAD on coronary CTA.

## Graphical Abstract

**Graphical Abstract:**
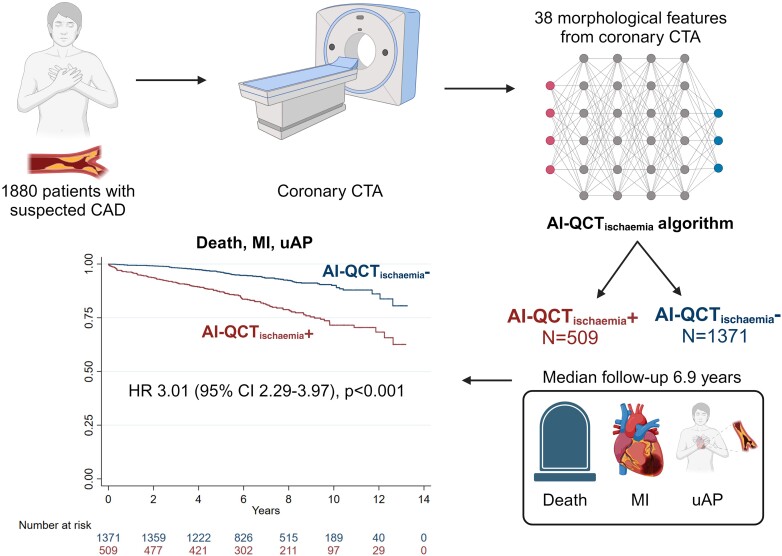
AI-QCT_ischaemia_− denotes normal AI-QCT_ischaemia_ result, and AI-QCT_ischaemia_+ denotes abnormal AI-QCT_ischaemia_ result. AI-QCT, artificial intelligence-guided quantitative computed tomography; CAD, coronary artery disease; CTA, computed tomography angiography; MI, myocardial infarction; uAP, unstable angina pectoris (illustrated with BioRender.com).

## Introduction

Coronary computed tomography angiography (CTA) has emerged as the first-line non-invasive imaging tool for the suspicion of coronary artery disease (CAD) amongst patients with low to intermediate pre-test probability of CAD.^1,2^ However, there is often disagreement between the anatomic and functional severity of CAD^3–6^ and coronary revascularization generally mandates the presence of ischaemia,^1,2^ which cannot directly be assessed by coronary CTA. Thus, a second imaging modality to determine myocardial ischaemia [e.g. positron emission tomography (PET), single-photon emission tomography, cardiac resonance imaging, and stress echocardiography] is recommended by current guidelines (Class IB^1^/Class IIaB^2^). However, using a second imaging modality after coronary CTA is associated with increased resource use and, depending on local availabilities, varying time delay until established diagnosis or decision on further management.

Artificial intelligence (AI)-based methods in cardiovascular imaging are increasingly being investigated and adopted to improve disease classification, reduce resource use, and improve prognostic power.^7,8^ The focus of this study is a novel AI-guided quantitative computed tomography ischaemia algorithm (AI-QCT_ischaemia_), comprising a machine-learned method that leverages features of atherosclerosis and vascular morphology from coronary CTA images to identify whether coronary lesions will likely cause myocardial ischaemia.^9–11^ With the addition of AI-QCT_ischaemia_ to standard coronary CTA, information on both anatomical and functional significance of CAD may be gained from a single coronary CTA scan. AI-QCT_ischaemia_, therefore, carries the potential to help refine risk stratification. Using our prospective patient cohort, we investigated the prognostic value of AI-QCT_ischaemia_ for adverse clinical events amongst a real-world population of patients with suspected CAD undergoing clinically indicated coronary CTA.

## Methods

### Patient population and follow-up

**Figure 1:**
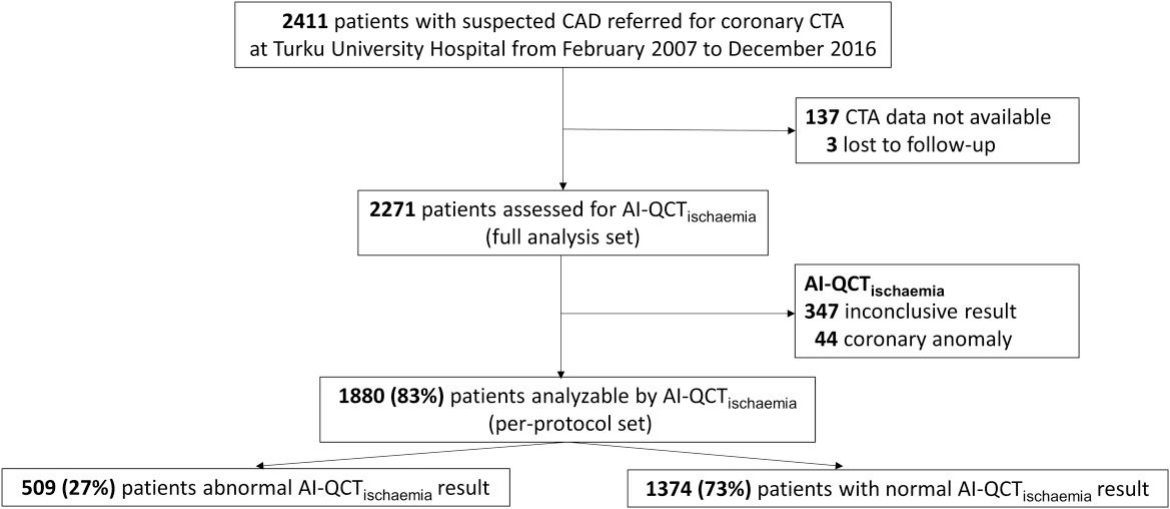
Patient flowchart. AI-QCT, artificial intelligence-guided quantitative computed tomography; CAD, coronary artery disease; CTA, computed tomography angiography.

A total of 2411 patients with suspected CAD underwent coronary CTA for suspected CAD at the Turku University Hospital from February 2007 to December 2016. Out of these, 2274 patients had coronary CTA image data available for AI-QCT_ischaemia_ analysis. Three patients were lost to follow-up, resulting in the final cohort of 2271 patients (*Figure 1*). Patients with previous myocardial revascularization or documented obstructive CAD (i.e. >50% diameter stenosis by invasive angiography) were not considered for inclusion. Data on clinical characteristics, symptoms, and medication were retrospectively collected from electronic medical records. Comprehensive data on all-cause death, myocardial infarction (MI), and unstable angina pectoris (uAP) were recorded using the registries of the Finnish National Institute for Health and Welfare and the Centre for Clinical Informatics of the Turku University Hospital. The events identified from the registries were confirmed by investigators using electronic medical records.

### Consent

The study complies with the Declaration of Helsinki. The Ethics Committee of the Hospital District of Southwest Finland approved the study protocol and waived the need for written informed consent.

### Coronary CTA imaging

Coronary CTA imaging was performed as described previously.^12^ In brief, coronary CTA scans were performed with at least 64-row hybrid PET-CT scanner (GE Discovery VCT or GE D690, GE Discovery MI, General Electric Medical Systems, Waukesha, USA). Before coronary CTA image acquisition, intravenous metoprolol (0–30 mg) to achieve a target heart rate of 60 bpm and isosorbide dinitrate aerosol (1.25 mg) or sublingual nitrate (800 µg) were administered. Coronary CTA was performed using intravenously administered low-osmolal iodine contrast agent. Prospectively triggered acquisition was applied whenever feasible.

### AI-QCT_ischaemia_ algorithm

Coronary CTA scans were initially analysed in a blinded manner using a previously described AI-QCT algorithm (Cleerly LABS, Cleerly Inc., Denver, CO, USA).^13,14^ This commercially available FDA-cleared software utilizes a series of validated convolutional neural networks (3D U-Net and VGG network variants) for image quality assessment, coronary segmentation and labelling, lumen wall evaluation and vessel contour determination, and plaque characterization. The quantitative output of the AI-QCT algorithm consists of presence or absence of features of (i) stenosis parameters, such as diameter stenosis (%) and area stenosis (%), number of severe stenosis (>70%), and number of moderate stenosis (50–70%); (ii) atherosclerosis measurements, such as non-calcified plaque volume, total plaque volume, and lesion length; (iii) vascular morphology features such as total vessel volume, total lumen volume, and vessel length; and (iv) diffuseness that includes a calculation of the sum of volumes and lengths across all involved segments. The AI-QCT results are then fed into an AI-QCT_ischaemia_ algorithm (Cleerly ISCHEMIA, Cleerly Inc., Denver, CO, USA; FDA cleared).^9^ AI-QCT_ischaemia_ is a method to determine the probability of myocardial ischaemia from coronary CTA data using a random forest machine-learned algorithm. AI-QCT_ischaemia_ determines the probability of abnormal invasive fractional flow reserve (FFR) using 38 CCTA-derived quantitative variables from Cleerly LABS. The output is binary, i.e. normal or abnormal AI-QCT_ischaemia_ result (*Graphical Abstract*). AI-QCT_ischaemia_ was trained against invasive FFR with cut-off 0.80,^9,15^ using data from the CREDENCE (*Computed tomogRaphic Evaluation of atherosclerotic DEtermiNants of myocardial isChEmia*)^16^ trial (50:50 derivation and internal validation) and has undergone external validation using data from the PACIFIC (*Prospective Comparison of Cardiac PET/CT, SPECT/CT Perfusion Imaging and CT Coronary Angiography With Invasive Coronary Angiography*)^17^ trial.

For the current study, patients were classified as having an abnormal AI-QCT_ischaemia_ result in case ≥1 analysable vessel was ischaemic based on the algorithm. Patients were classified as having a normal AI-QCT_ischaemia_ result in case all three main vessels [left anterior descending artery, left circumflex artery (including also left main), and right coronary artery] were analysable and non-ischaemic according to the algorithm. If there was ≥1 non-evaluable vessel in the absence of any ischaemic vessels, patients were considered as inconclusive by AI-QCT_ischaemia_ and excluded from the per-protocol analysis. In the full-analysis set, these patients were assessed in an intention-to-diagnose approach, i.e. by classifying those with an inconclusive AI-QCT_ischaemia_ result or those excluded due to coronary anomalies as having an abnormal AI-QCT_ischaemia_ result.

### Primary endpoint and study aims

The primary endpoint was the composite of death, MI, or uAP. The primary aim of this analysis was to assess the rate of the primary endpoint amongst patients with abnormal vs. normal AI-QCT_ischaemia_ result. The secondary aim was to assess the primary endpoint separately amongst patients with no/non-obstructive CAD (i.e. visual diameter stenosis ≤ 50%) and patients with obstructive CAD (visual diameter stenosis > 50%) in order to evaluate the prognostic value of AI-QCT_ischaemia_ amongst two patient subgroups that are currently managed differently (i.e. non-obstructive disease with medical treatment and obstructive disease with referral for functional testing and/or invasive angiography to assess the need for revascularization). MIs were type 1 MIs^18^ and uAP was defined according to the clinical definition^19^ and an acute plaque event confirmed by invasive angiography.

### Statistical analysis

The statistical analyses were performed in an independent academic setting at the Turku University Hospital.

Continuous variables are shown as mean ± SD or median [interquartile range (25th–75th percentile)]. Categorical variables are shown as numbers with percentages. Mann–Whitney *U* test was used to compare continuous variables and two-sided chi-square test was used for categorical variables. Kaplan–Meier curves for clinical events were created and compared with the log-rank test between patients with abnormal vs. normal AI-QCT_ischaemia_ result. We report crude and adjusted event rates from multivariable Cox proportional hazard models. Adjusting covariates were chosen based on clinical reasoning and consisted of age, sex, hypertension, diabetes mellitus, smoking, dyslipidaemia, family history of CAD, and typical angina. Variables with a significant association in univariable models were included into the multivariable models. We also compared three multivariable Cox models for the primary endpoint with the following covariates: clinical model (1): age, sex, hypertension, diabetes, smoking, typical angina (based on significant univariable associations with the primary endpoint); clinical + stenosis model (2): with added visual obstructive stenosis >50%; and clinical + stenosis + AI-QCT_ischaemia_ model (3): with added AI-QCT_ischaemia_. We subsequently ran Models 1 and 3 stratified according to the presence of obstructive stenosis and sex. The prognostic performance of the models was compared with Harrel’s C. Prognostic modelling with AI-QCT stenosis and AI-QCT_ischaemia_ combined was not feasible due to multi-collinearity (variance inflation factors > 5). Analyses were two-tailed and a *P*-value of <0.05 was considered statistically significant. All analyses were performed in Stata version 15 (StataCorp. 2017. Stata Statistical Software: Release 15. College Station, TX, USA: StataCorp LLC).

## Results

### Patient population

Out of the cohort of 2271 patients, 1880 (83%) patients had conclusive AI-QCT_ischaemia_ result; 509/1880 (27.1%) patients had abnormal and 1371/1880 (72.9%) patients had normal AI-QCT_ischaemia_ result (*Figure 1*). The decision on revascularization was based on functionally significant stenosis according to 15-O-water PET perfusion or invasive FFR. Out of 1880 patients, 662 (35.2%) were referred for 15-O-water PET perfusion, and subsequently 413 (22.0%) of the patients were referred for invasive coronary angiography and 204 (10.9%) underwent early elective revascularization within 6 months. Since for most patients undergoing coronary angiography, the PET perfusion result was available, the use of invasive FFR was not systematically collected. Patients with abnormal AI-QCT_ischaemia_ result were significantly older, more frequently male, had more frequently hypertension, diabetes mellitus, dyslipidaemia, smoking history, typical angina pectoris, and higher New York Heart Association (NYHA) class, and were more frequently on an antiplatelet agent, oral anticoagulation, lipid-lowering, anti-anginal, and/or anti-hypertensive drug. Agatston Coronary Calcium Score and visual diameter stenosis were remarkably higher in patients with abnormal vs. normal AI-QCT_ischaemia_ result (*Table 1*). Patients with an abnormal AI-QCT_ischaemia_ result were also more frequently referred for downstream 15-O-water PET perfusion imaging and invasive coronary angiography; 36.0% of the patients with abnormal AI-QCT_ischaemia_ result as compared with 1.5% of patients with normal AI-QCT_ischaemia_ result underwent early elective revascularization (*P* < 0.001) (*Table 1*).

**Table 1:** Patient baseline characteristics

	*n*	Abnormal	*n*	Normal	*P*-value
		AI-QCT_ischaemia_ result (*n* = 509)		AI-QCT_ischaemia_ result (*n* = 1371)	
Age, years	509	67 [61–72]	1371	62 [55–68]	<0.001
Sex (female), *n* (%)	509	200 (38.3%)	1371	846 (61.7%)	<0.001
Hypertension, *n* (%)	509	352 (69.2%)	1371	707 (51.6%)	<0.001
Dyslipidaemia, *n* (%)	509	370 (72.7%)	1371	848 (61.9%)	<0.001
Current smoker, *n* (%)	509	74 (14.5%)	1371	165 (12.0%)	0.148
Previous smoker, *n* (%)	509	138 (27.1%)	1371	256 (18.7%)	<0.001
BMI, kg/m^2^	435	27.8	773	27.4	0.549
		[24.8–30.8]		[24.6–31.1]	
Diabetes mellitus, *n* (%)	509	109 (21.4%)	1371	180 (13.1%)	<0.001
Pre-diabetes^a^, *n* (%)	509	92 (18.1%)	1371	176 (12.8%)	0.004
Family history of CAD, *n* (%)	509	228 (44.8%)	1371	660 (48.1%)	0.197
Typical angina, *n* (%)	509	160 (31.4%)	1371	283 (20.6%)	<0.001
NYHA class					
I		153 (48.0%)		547 (65.1%)	
II	319	140 (43.9%)	840	271 (32.3%)	<0.001
III		26 (8.1%)		22 (2.6%)	
Visual diameter stenosis, *n* (%)					
0%		1 (0.2%)		542 (40.0%)	
1–50%	491	112 (22.8%)	1356	718 (53.0%)	<0.001
>50%		378 (77.0%)		96 (7.0%)	
Agatston Coronary	416	500	1133	6	
Calcium Score		[242–1215]		[0–87]	
Downstream PET performed, *n* (%)	509	416 (81.7%)	1371	246 (17.9%)	<0.001
Elective referral for ICA (within 6 months), *n* (%)	509	268 (52.7%)	1371	145 (10.6%)	<0.001
Early revascularization (within 6 months, PCI or CABG), *n* (%)	509	183 (36.0%)	1371	21 (1.5%)	<0.001
Early PCI (within 6 months), *n* (%)	509	149 (29.3%)	1371	20 (1.5%)	<0.001
Early CABG (within 6 months), *n* (%)	509	38 (7.5%)	1371	2 (0.1%)	<0.001
Antiplatelet drug	509	283 (55.6%)	1371	555 (40.5)	<0.001
(Aspirin or other), *n* (%)					
Anticoagulation, *n* (%)	509	48 (9.4%)	1371	90 (6.6%)	0.034
Lipid-lowering drug, *n* (%)	509	277 (54.4%)	1371	506 (36.9%)	<0.001
Beta-blocker, *n* (%)	509	272 (53.4%)	1371	558 (40.7%)	<0.001
Long-acting nitrate, *n* (%)	509	55 (10.8%)	1371	99 (7.2%)	0.012
Calcium channel blocker, *n* (%)	509	105 (20.6%)	1371	163 (11.9%)	<0.001
ACE inhibitor, *n* (%)	509	114 (22.4%)	1371	206 (15.0%)	<0.001
AT II antagonist, *n* (%)	509	122 (24.0%)	1371	263 (19.2%)	0.022
Diuretic, *n* (%)	509	120 (23.6%)	1371	215 (15.7%)	<0.001
Antiarrhythmic drug, *n* (%)	509	13 (2.6%)	1371	33 (2.4%)	0.855

Values are *n* (%) or mean [±standard deviation (SD)] or median [interquartile range]. *P*-values are from Mann–Whitney *U* tests or chi-square tests.

AI-QCT, artificial intelligence-guided quantitative computed tomography; ACE, angiotensin converting enzyme; AP, angina pectoris; AT II, angiotensin II; BMI, body mass index; CABG, coronary artery bypass grafting; CAD, coronary artery disease; FFR, fractional flow reserve; ICA, invasive coronary angiography; NYHA, New York Heart Association; PCI, percutaneous coronary intervention; PET, positron emission tomography.

^a^Pre-diabetes was defined as HbA1c 6.0–6.5%, or fasting glucose 6.1–6.9 mmol/L or impaired glucose tolerance (2-h plasma glucose 7.8–11.0 mmol/L in a 75 oral glucose tolerance test).

### Primary endpoint

**Figure 2:**
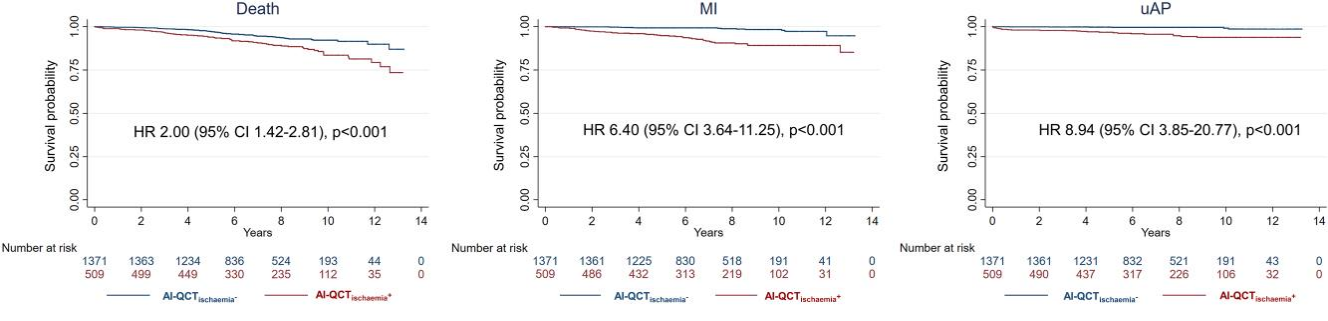
Kaplan–Meier curves of the primary endpoint components. Crude Kaplan–Meier curves and HRs of the primary endpoint composites death, MI, and uAP. AI-QCT_ischaemia_− denotes normal AI-QCT_ischaemia_ result and AI-QCT_ischaemia_+ denotes abnormal AI-QCT_ischaemia_ result. AI-QCT, artificial intelligence-guided quantitative computed tomography; CI, confidence interval; HR, hazard ratio; MI, myocardial infarction; uAP, unstable angina pectoris.

The follow-up time for the primary endpoint was median 6.9 [interquartile range 4.8–9.0] years. Patients with an abnormal AI-QCT_ischaemia_ result (27.1%, *n* = 509/1880) as compared with patients with a normal AI-QCT_ischaemia_ result (72.9%, *n* = 1371/1880) had a significantly higher crude rate of the primary endpoint [hazard ratio (HR) 3.01, 95% confidence interval (CI) 2.29–3.97, *P* < 0.001] (*Graphical Abstract*; *Table 2*), driven by significantly higher rates of all primary endpoint components (death: HR 2.00, 95% CI 1.42–2.81, *P* < 0.001; MI: HR 6.50, 95% CI 3.64–11.25, *P* < 0.001; uAP: HR 8.94, 95% CI 3.85–20.77, *P* < 0.001) (*Figure 2*; *Table 2*). Age, sex, hypertension, diabetes, smoking, and typical angina showed significant associations with the primary endpoint composite in univariable Cox regressions (see Supplementary data online, *Table S1*) and were used as adjusting covariates in the multivariable analysis. Results of this adjusted analysis remained consistent (primary endpoint adjusted HR (HR_adj_) 1.96, 95% CI 1.46–2.63, *P* < 0.001). Adjusted analyses also showed consistent results for MI (HR_adj_ 4.61, 95% CI 2.56–8.28, *P* < 0.001), and uAP (HR_adj_ 6.52, 95% CI 2.74–15.50, *P* < 0.001), but statistical significance was lost for death alone (HR_adj_ 1.27, 95% CI 0.89–1.82, *P* = 0.182) (*Table 2*; Supplementary data online, *Table S1*).

**Table 2:** Per-protocol set

All patients (*n* = 1880)	Crude hazard ratios				Adjusted hazard ratios			
	Abnormal	Normal	HR (95% CI)	*P*-value	*n* patients	*n* events	HR (95% CI) adjusted	*P*-value adjusted
	AI-QCT_ischaemia_ result (*n* = 509)	AI-QCT_ischaemia_ result (*n* = 1371)						
Death, MI, or uAP, *n* (%)	110 (21.6%)	96 (7.0%)	3.01 (2.29–3.97)	<0.001	1880	206	1.96 (1.46–2.63)	<0.001^a^
Death, *n* (%)	61 (12.0%)	74 (5.4%)	2.00 (1.42–2.81)	<0.001	1880	135	1.27 (0.89–1.82)	0.182^b^
MI, *n* (%)	42 (8.3%)	17 (1.2%)	6.40 (3.64–11.25)	<0.001	1880	59	4.61 (2.56–8.28)	<0.001^c^
uAP, *n* (%)	24 (4.7%)	7 (0.5%)	8.94 (3.85–20.77)	<0.001	1880	31	6.52 (2.74–15.50)	<0.001^d^

Patients with visual diameter stenosis ≤ 50% (*n* = 1373)	Abnormal	Normal	HR (95% CI)	*P*-value	*n* patients	*n* events	HR (95% CI) adjusted	*P*-value adjusted
	AI-QCT_ischaemia_ result (*n* = 113)	AI-QCT_ischaemia_ result (*n* = 1260)						
Death, MI, or uAP, *n* (%)	20 (17.7%)	79 (6.3%)	2.86 (1.75–4.67)	<0.001	1373	99	1.81 (1.09–3.00)	0.022^c^
Death, *n* (%)	12 (10.6%)	61 (4.8%)	2.16 (1.16–4.02)	0.015	1373	73	1.53 (0.82–2.86)	0.185^e^
MI, *n* (%)	6 (5.3%)	13 (1.0%)	5.18 (1.97–13.62)	0.001	1373	19	4.79 (1.81–12.64)	0.002^f^
uAP, *n* (%)	3 (2.7%)	6 (0.5%)	5.48 (1.37–21.93)	0.016	1373	9	3.93 (0.96–16.00)	0.056^g^

Patients with visual diameter stenosis > 50% (*n* = 474)	Abnormal	Normal	HR (95% CI)	*P*-value	*n* patients	*n* events	HR (95% CI) adjusted	*P*-value adjusted
	AI-QCT_ischaemia_ result (*n* = 378)	AI-QCT_ischaemia_ result (*n* = 96)						
Death, MI, or uAP, *n* (%)	90 (23.8%)	17 (17.7%)	1.33 (0.79–2.24)	0.279	474	107	1.26 (0.75–2.12)	0.386^g^
Death, *n* (%)	49 (13.0%)	13 (13.5%)	0.89 (0.48–1.64)	0.700	474	62	0.83 (0.45–1.54)	0.557^h^
MI, *n* (%)	36 (9.5%)	4 (4.2%)	2.25 (0.80–6.32)	0.125	474	40	-	-
uAP, *n* (%)	21 (5.6%)	1 (1.0%)	5.29 (0.71–39.30)	0.104	474	22	-	-

Displayed are numbers (percentage) of first events and HRs with 95% CIs from Cox proportional hazards models. HRs were adjusted for covariates with significant univariable associations with the reported endpoints (see Supplementary data online, *Tables S1*, *S6*, and *S7*).

AI-QCT, artificial intelligence-guided quantitative computed tomography; CI, confidence interval; HR, hazard ratio; MI, myocardial infarction; uAP, unstable angina pectoris.

^a^Age, sex, hypertension, diabetes, smoking, and typical angina.

^b^Age, hypertension, diabetes, smoking, and family history of coronary artery disease (CAD).

^c^Age, hypertension, smoking, and typical angina.

^d^Age, hypertension, typical angina.

^e^Age and family history of CAD.

^f^Typical angina.

^g^Age.

^h^Age and diabetes.

The analysis was repeated using the full-analysis set (*n* = 2271) in an intention-to-diagnose approach [i.e. classifying those patients with inconclusive AI-QCT_ischaemia_ result or excluded patients due to coronary anomalies (*n* = 391) as having an abnormal AI-QCT_ischaemia_ result]. Results of the full-analysis set were consistent, except for the adjusted rate of death, which was significantly higher in patients with an abnormal as compared and a normal AI-QCT_ischaemia_ results (see Supplementary data online, *Tables S2* and *S3*).

C-indexes for three different Cox regressions for the primary endpoint were as follows: clinical model (1): 0.707; clinical + stenosis model (2): 0.736; and clinical + stenosis + AI-QCT_ischaemia_ model (3): 0.739. The improvement in C-index was statistically significant for Model 2 vs. Model 1 (*P* = 0.001) and Model 3 vs. Model 1 (*P* = 0.001), but not for Model 3 vs. Model 2 (*P* = 0.332). However, AI-QCT_ischaemia_ remained an independent predictor of the primary endpoint on top of visual stenosis and clinical factors in Model 3 (HR 1.52, 95% CI 1.03–2.25, *P* = 0.036) (*Table 3*). In stratified versions of Models 1 and 3 according to sex, AI-QCT_ischaemia_ remained an independent predictor on top of clinical variables amongst both sexes, but C-indexes were only non-significantly improved (see Supplementary data online, *Tables S4* and *S5*).

**Table 3:** Multivariable cox models and c-indexes for the primary endpoint

Death, MI, or uAP	Clinical model (1)		Clinical + stenosis model (2)		Clinical + stenosis + AI-QCT_ischaemia_ model (3)	
1847 patients	HR (95% CI)	*P*-value	HR (95% CI)	*P*-value	HR (95% CI)	*P*-value
206 events						
AI-QCT_ischaemia_	-	-	**-**	**-**	**1.52** (**1.03–2.25)**	**0**.**036**
Visual obstructive stenosis > 50%	**-**	**-**	**1.97** (**1.47–2.65)**	**<0**.**001**	**1.49** (**1.01–2.21)**	**0**.**045**
Age, per 1 year	**1.07** (**1.06–1.09)**	**<0**.**001**	**1.07** (**1.05–1.09)**	**<0**.**001**	**1.06** (**1.04–1.08)**	**<0**.**001**
Sex (male vs. female)	**1.48** (**1.11–1.97)**	**0**.**007**	1.27 (0.95–1.70)	0.104	1.24 (0.92–1.66)	0.153
Hypertension	1.35 (1.00–1.83)	0.050	1.29 (0.95–1.74)	0.099	1.27 (0.94–1.72)	0.116
Diabetes mellitus	1.32 (0.94–1.85)	0.104	1.19 (0.85–1.67)	0.310	1.20 (0.86–1.69)	0.285
Smoker	**1.67** (**1.25–2.22)**	**<0**.**001**	**1.59** (**1.20–2.12)**	**0**.**001**	**1.57** (**1.18–2.09)**	**0**.**002**
Typical angina	**1.42** (**1.06–1.91)**	**0**.**019**	1.31 (0.97–1.76)	0.079	1.29 (0.95–1.73)	0.098
C-index (95% CI)	**0.707** (**0.672–0.742)**	**<0**.**001**	**0.736** (**0.702–0.769)**	**<0**.**001**	**0.739** (**0.706–0.773)**	**<0**.**001**
C-index difference (95% CI)	**Model 2-Model 1**	***P*-value**	**Model 3-Model 2**	***P*-value**	**Model 3-Model 1**	***P*-value**
	**0.029** (**0.011–0.046)**	**0**.**001**	0.003 (−0.004–0.011)	0.332	**0.032** (**0.013–0.051)**	**0**.**001**

Displayed are HRs with 95% CIs from multivariable Cox regressions, C-index per model, and difference in C-indexes between the models. The clinical variables were chosen based on significant univariable associations with the primary endpoint (see Supplementary data online, *Table S1*). The sample size is given by the number of patients with available visual stenosis reading (*n* = 1847). Statistically significant associations (*P*-value < 0.05) are displayed in bold.

AI-QCT, artificial intelligence-guided quantitative computed tomography; CI, confidence interval; HR, hazard ratio; MI, myocardial infarction; uAP, unstable angina pectoris.

### Primary endpoint amongst patients with ≤50% or >50% visual diameter stenosis

Visual stenosis reading was available for 1847/1880 (98.2%) of the per-protocol population. Overall, 1373/1847 (74.3%) of patients had no/non-obstructive CAD on coronary CTA (i.e. ≤50% visual diameter stenosis) and 474/1847 (25.7%) had obstructive CAD on coronary CTA (i.e. >50% visual diameter stenosis). Patient baseline characteristics, clinical management, and medication use according to the AI-QCT_ischaemia_ result for patients with no/non-obstructive or obstructive CAD are shown in Supplementary data online, *Tables S6* and *S7*.

**Figure 3:**
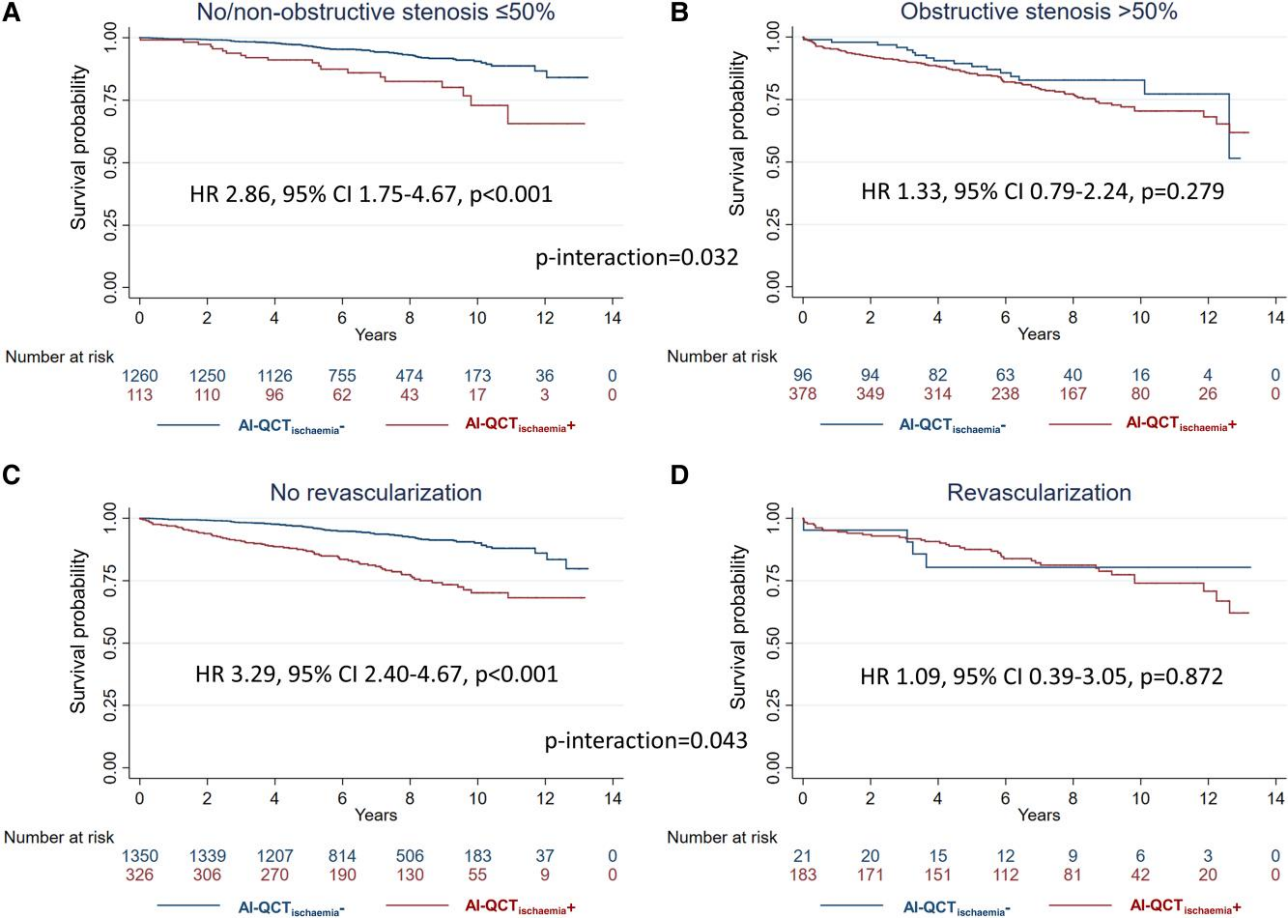
Subgroup analyses visual diameter stenosis and early revascularization. Crude Kaplan–Meier curves and HRs for the primary endpoint (death, myocardial infarction, unstable angina pectoris) are shown amongst patients with ≤50% (*A*) vs. >50% (*B*) visual diameter stenosis, as well as without early revascularization (*C*) vs. with early revascularization (*D*) within 6 months from coronary computed tomography angiography. AI-QCT_ischaemia_− denotes normal AI-QCT_ischaemia_ result and AI-QCT_ischaemia_+ denotes abnormal AI-QCT_ischaemia_ result. AI-QCT, artificial intelligence-guided quantitative computed tomography; CI, confidence interval.

Amongst the 1373 patients with no/non-obstructive CAD, AI-QCT_ischaemia_ result was normal in 1260 (91.8%) and abnormal in 113 (8.2%) patients. In contrast, amongst the 474 patients with obstructive CAD on coronary CTA, AI-QCT_ischaemia_ result was normal in 96 (20.3%) and abnormal in 378 (79.7%) patients. An abnormal AI-QCT_ischaemia_ result as compared with a normal AI-QCT_ischaemia_ result was associated with a significantly higher crude rate of the primary endpoint amongst patients with no/non-obstructive CAD (HR 2.86, 95% CI 1.75–4.67, *P* < 0.001) but not amongst those with obstructive CAD (HR 1.33, 95% CI 0.79–2.24, *P* = 0.279; *P*-interaction = 0.032) (*Figure 3A* and *B*; *Table 2*). According to the univariable Cox regressions (see Supplementary data online, *Tables S8* and *S9*), age, hypertension, smoking, and typical angina were used as adjusting covariates for the subgroup with no/non-obstructive CAD and age for the subgroup with obstructive CAD. Results of the adjusted analyses remained consistent (*Table 2*).

Amongst patients with no/non-obstructive disease, C-index of the clinical model (1) was 0.722 and for the clinical + AI-QCT_ischaemia_ model (2) 0.726. This improvement in C-index was statistically not significant (*P* = 0.535), but AI-QCT_ischaemia_ remained an independent predictor of the primary endpoint (HR 1.74, 95% CI 1.04–2.90, *P* = 0.035) (see Supplementary data online, *Table S10*). Amongst patients with obstructive disease, C-index of the clinical model (1) was 0.602, similar to that of the clinical + AI-QCT_ischaemia_ model (2) of 0.604 (*P* = 0.741). However, in model 2, AI-QCT_ischaemia_ was no independent predictor of the primary endpoint (HR 1.27, 95% CI 0.75–2.14, *P* = 0.374) (see Supplementary data online, *Table S11*).

### Subgroup analysis for patients with vs. without early revascularization

Amongst the 1676 patients not undergoing early revacularization, 326 (19.5%) patients had an abnormal, 1350 (80.5%) patients had a normal AI-QCT_ischaemia_ result, and there was incremental risk stratification for the primary endpoint by AI-QCT_ischaemia_ (HR 3.29, 95% CI 2.40–4.67, *P* < 0.001) (*Figure 3C*). Contrarily, amongst the 204 patients who underwent revascularization, 183 (89.7%) had an abnormal, 21 (10.3%) had a normal AI-QCT_ischaemia_ result, and AI-QCT_ischaemia_ showed no incremental risk stratification (HR 1.09, 95% CI 0.39–3.05, *P* = 0.872) (*P*-interaction = 0.043) (*Figure 3D*). These results also remained consistent in adjusted analyses based on significant univariable associations (see Supplementary data online, *Table S12*) with the primary endpoint (no revascularization group: HR_adj_ 2.06, 95% CI 1.48–2.85, *P* < 0.001; covariates: age, sex, hypertension, diabetes, smoking, and typical angina; revascularization group: HR_adj_ 0.87, 95% CI 0.31–2.49, *P* = 0.802; covariate: age).

## Discussion

We re-analysed our prospective patient cohort to assess the long-term prognostic value of a novel AI-based coronary CTA-derived ischaemia algorithm in a real-world population with suspected CAD undergoing clinically indicated coronary CTA. The salient findings of this study can be summarized as follows: (i) calculation of AI-QCT_ischaemia_ was feasible from real-world CTA data in 83% of patients; (ii) an abnormal AI-QCT_ischaemia_ result was associated with a two-fold adjusted rate of all-cause death, MI, or uAP throughout median 7 years of follow-up, driven by higher rates of MI and uAP; and (iii) AI-QCT_ischaemia_ remained an independent predictor of the primary endpoint on top of visual stenosis and clinical factors, and the risk differentiation was most prominent amongst patients with no or non-obstructive CAD (i.e. ≤50% visual diameter stenosis).

### Functional assessment of CAD from coronary CTA

The use of coronary CTA as the initial diagnostic tool for the suspicion of CAD amongst symptomatic patients with low to intermediate pre-test probability of CAD has shown to be feasible,^20–22^ improves the 5-year risk of death from CAD or MI related to a higher prescription rate of lipid-lowering and/or antiplatelet drugs,^23^ and is therefore recommended by current guidelines.^1,2^ However, there is often disagreement between the anatomic and functional severity of CAD,^3–6^ and the indication for myocardial revascularization generally mandates the proof of ischaemia, unless a very high degree of stenosis (i.e. >90%) is present.^1,2^

Other approaches to pair anatomical and functional information from coronary CTA directly have been developed and consist currently of myocardial CT perfusion (CTP) imaging^24^ and CT-derived FFR (CT-FFR).^25^ Both modalities in combination with coronary CTA have shown to improve the specificity of detecting haemodynamically significant CAD as compared with coronary CTA alone.^26^ Also, their prognostic performance for clinical events has been shown up to 5 years for CT-FFR^27,28^ and 1.5 years for myocardial CTP.^29,30^ However, the main disadvantages of myocardial CTP are the need for additional specific CT image acquisition, thus higher doses of radiation, and the infusion of a hyperaemic agent.^31^ CT-FFR is calculated during CT image post-processing applying fluid dynamic equations and is dependent on optimal coronary CTA image quality,^25^ which was generally achievable in 85–90% of patients in study settings.^32,33^ CT-FFR being recommended by societal guidelines^2,34^ is already used in clinical practice. However, intention-to-diagnose analyses^35^ and real-world data^36,37^ have shown substantially lower test characteristics and more difficulties with image quality leading to impossible CT-FFR calculation of at least 1 vessel in 25% of patients.^35^ Thus, there is room for improved or novel approaches to determine coronary ischaemia from coronary CTA data.

### AI-based coronary CTA-derived analysis of myocardial ischaemia

AI-based analyses for the assessment of CAD are increasingly being developed,^7,8^ and they may revolutionize image analysis in the future. In this regard, we tested the prognostic value of a recently developed AI-algorithm based on morphology from coronary CTA images to identify whether coronary lesions will likely cause myocardial ischaemia. Our analysis shows that AI-QCT_ischaemia_ calculation was feasible from coronary CTA data obtained during the clinical routine in 83% of cases, which is comparable with CT-FFR.^32,33^ AI-QCT_ischaemia_ also proved its prognostic power with a two-fold increased adjusted long-term rate of the primary endpoint. This is consistent with previous data on quantitative PET perfusion.^38^ Furthermore, C-index improvement with the addition of AI-QCT_ischaemia_ was significant in comparison with clinical variables and similar to that of visual obstructive stenosis. However, AI-QCT_ischaemia_ remained an independent predictor of the primary endpoint on top of clinical variables and visual stenosis.

The second aim of this investigation was to determine whether AI-QCT_ischaemia_ had differential risk stratification amongst patients with no/non-obstructive or obstructive CAD. First of all, in this analysis, 79.8% of patients with >50% stenosis had ‘ischaemia’ according to AI-QCT_ischaemia_. This is a substantially higher proportion of patients having ‘ischaemia’ as commonly reported, where agreement between anatomically obstructive disease and (invasively or non-invasively determined) ischaemia is usually 30–47%.^3,5,6^ This may be explained by the fact that diameter stenosis is the highest ranked feature in the AI-QCT_ischaemia_ algorithm, and it has been reported previously that obstructive disease according to AI-QCT diameter stenosis is related to ischaemia in up to 76% of the cases.^39^ Despite using visual and not AI-QCT stenosis, it appears plausible that the higher classification agreement between obstructive and ‘functionally’ significant disease according to AI-QCT_ischaemia_ is related to the high feature importance of stenosis in the AI-QCT_ischaemia_ algorithm.

Amongst patients with non-obstructive disease, we found incremental risk stratification by AI-QCT_ischaemia_, and although sample size and event numbers are small and CIs wide, the results indicate that this risk difference was driven by a significantly higher adjusted rate of spontaneous MI, whereas adjusted mortality rates were similar. Revascularization was performed in <1% in this patient group with non-obstructive disease (in 5.3% of patients with abnormal AI-QCT_ischaemia_ and 0.3% with normal AI-QCT_ischaemia_ result). These results are in line with current evidence that ischaemia based on contemporary non-invasive methods is associated with an increased risk of spontaneous MI if managed medically,^40,41^ whereas all-cause mortality is similar amongst revascularized vs. medically treated patients with ischaemic CAD.^42–44^ Vice versa, the absence of incremental risk stratification by AI-QCT_ischaemia_ amongst patients with obstructive disease could be explained by the 39.7% of patients who underwent early revascularization (45.5% of patients with abnormal AI-QCT_ischaemia_ and 16.7% with normal AI-QCT_ischaemia_ result). These considerations are also supported by the subgroup analysis in the total cohort for patients with vs. without early revascularization, where incremental risk stratification by AI-QCT_ischaemia_ was only found amongst those without revascularization. However, these are observations from a *post hoc* analysis of cohort data and only an adequate, prospective, randomized-controlled trial could demonstrate the effect of revascularization amongst patients with an abnormal AI-QCT_ischaemia_ result. Nevertheless, the findings highlight the utility of a test to determine the haemodynamic consequences of CAD also in the non-obstructive range, and the results are also in agreement with earlier findings that non-obstructive CAD on coronary CTA is associated with increased overall event risk.^23,45–47^

Together with its good calculability from real-world clinical coronary CTA data, as well as radiation and hyperaemia-free nature, AI-QCT_ischaemia_ may thus represent a promising tool for improved risk stratification amongst patients with coronary atherosclerosis and may become an alternative to other functional coronary CTA tests consisting to date of myocardial CTP and CT-FFR. Further research on its agreement with other ischaemia tests and prognostic power in different patient populations is warranted.

## Limitations

The results of this study must be considered in the light of several limitations: (i) it was a single-centre re-analysis of observational study cohort data; (ii) in 17% of the patients, AI-QCT_ischaemia_ was inconclusive or not analysable (the latter due to coronary anomalies); however, we have included these patients in an intention-to-diagnose approach; (iii) the algorithm is binary and does currently not quantify the magnitude of ischaemia; and (iv) subgroups (≤50% visual diameter stenosis with abnormal AI-QCT_ischaemia_ result, *n* = 113; >50% visual diameter stenosis with normal AI-QCT_ischaemia_ result, *n* = 96) were relatively small, which hampers the evaluation of different event types (death vs. MI vs. uAP).

## Conclusions

Amongst patients with suspected CAD undergoing clinically indicated coronary CTA, an abnormal AI-QCT_ischaemia_ result was associated with a two-fold increased adjusted rate of long-term death, MI, or uAP. AI-QCT_ischaemia_ may thus be useful to improve risk stratification, especially amongst patients with no/non-obstructive CAD on coronary CTA.

## Supplementary data

Supplementary data are available at *European Heart Journal - Cardiovascular Imaging* online.

## Supplementary Material

jead339_Supplementary_Data

## Data Availability

The data underlying this article will be shared on reasonable request from the corresponding author.
