# Supplementary material for: Prognostic value of a novel artificial intelligence-based coronary computed tomography angiography-derived ischaemia algorithm for patients with suspected coronary artery disease
Source: Eur Heart J Cardiovasc Imaging. 2023 Dec 12;25(5):657–67. doi: 10.1093/ehjci/jead339 (PMC11057943; doi:10.1093/ehjci/jead339)
Supplement: jead339_Supplementary_Data [file jead339_supplementary_data.docx]

**SUPPLEMENTARY MATERIAL**

**Prognostic Value of a Novel Artificial Intelligence-Based Coronary Computed Tomography Angiography-Derived Ischemia Algorithm for Patients with Suspected Coronary Artery Disease**

**Table S1. Univariable Cox Regressions Per-Protocol-Set**

| **N=1880** | **Death, MI, or uAP**  **206 events** | | **Death**  **135 events** | | **MI**  **59 events** | | **uAP**  **31 events** | |
| --- | --- | --- | --- | --- | --- | --- | --- | --- |
|  | **HR (95% CI)** | **p-value** | **HR (95% CI)** | **p-value** | **HR (95% CI)** | **p-value** | **HR (95% CI)** | **p-value** |
| Age, per 1 year | **1.07 (1.05-1.09)** | **<0.001** | **1.08 (1.06-1.10)** | **<0.001** | **1.05 (1.02-1.09)** | **0.001** | **1.06 (1.01-1.10)** | **0.010** |
| Sex  (male vs. female) | **1.42 (1.08-1.87)** | **0.012** | 1.28 (0.91-1.80) | 0.151 | 1.51 (0.90-2.53) | 0.116 | 1.46 (0.72-2.97) | 0.291 |
| Hypertension | **1.72 (1.28-2.30)** | **<0.001** | **1.50 (1.05-2.13)** | **0.026** | **2.16 (1.22-3.83)** | **0.009** | **3.33 (1.37-8.13)** | **0.008** |
| Diabetes mellitus | **1.68 (1.21-2.34)** | **0.002** | **2.05 (1.39-3.01)** | **<0.001** | 1.65 (0.89-3.06) | 0.110 | 0.39 (0.09-1.65) | 0.201 |
| Smoker | **1.41 (1.09-1.82)** | **0.008** | **1.58 (1.13-2.22)** | **0.008** | **1.90 (1.14-3.17)** | **0.014** | 1.39 (0.68-2.85) | 0.362 |
| Dyslipidemia | 0.94 (0.71-1.26) | 0.688 | 0.81 (0.57-1.15) | 0.237 | 1.84 (0.99-3.40) | 0.053 | 1.11 (0.52-2.35) | 0.791 |
| Family history  of CAD | 0.83 (0.63-1.09) | 0.183 | **0.63 (0.44-0.89)** | **0.010** | 1.22 (0.73-2.04) | 0.439 | 1.35 (0.66-2.73) | 0.411 |
| Typical angina | **1.57 (1.17-2.11)** | **0.003** | 1.05 (0.71-1.55) | 0.815 | **2.29 (1.36-3.85)** | **0.002** | **2.77 (1.36-5.62)** | **0.005** |

Displayed are hazard ratios (HR) with 95% confidence intervals (CI) from univariable Cox proportional hazards models. CAD = coronary artery disease, MI = myocardial infarction, uAP = unstable angina pectoris.

**Table S2. Univariable Cox Regressions Full-Analysis-Set**

| **N=2271** | **Death, MI, or uAP**  **255 events** | | **Death**  **173 events** | | **MI**  **68 events** | | **uAP**  **34 events** | |
| --- | --- | --- | --- | --- | --- | --- | --- | --- |
|  | **HR (95% CI)** | **p-value** | **HR (95% CI)** | **p-value** | **HR (95% CI)** | **p-value** | **HR (95% CI)** | **p-value** |
| Age, per 1 year | **1.07 (1.05-1.09)** | **<0.001** | **1.09 (1.07-1.11)** | **<0.001** | **1.05 (1.02-1.08)** | **<0.001** | **1.05 (1.01-1.09)** | **0.013** |
| Sex  (male vs. female) | **1.44 (1.12-1.84)** | **0.004** | 1.30 (0.96-1.75) | 0.089 | 1.57 (0.98-2.54) | 0.063 | 1.69 (0.86-3.33) | 0.129 |
| Hypertension | **1.78 (1.36-2.31)** | **<0.001** | **1.60 (1.17-2.20)** | **0.004** | **2.19 (1.28--3.75)** | **0.004** | **3.03 (1.32-6.96)** | **0.009** |
| Diabetes mellitus | **1.61 (1.19-2.17)** | **0.002** | **1.87 (1.32-2.64)** | **<0.001** | 1.50 (0.83-2.70) | 0.176 | 0.55 (0.17-1.80) | 0.325 |
| Smoker | **1.44 (1.12-1.85)** | **0.004** | **1.50 (1.11-2.02)** | **0.009** | **1.71 (1.06-2.75)** | **0.028** | 1.24 (0.62-2.48) | 0.541 |
| Dyslipidemia | 0.98 (0.75-1.26) | 0.851 | 0.88 (0.65-1.20) | 0.437 | **1.76 (1.00-3.09)** | **0.047** | 1.01 (0.50-2.04) | 0.980 |
| Family history  of CAD | 0.84 (0.65-1.07) | 0.159 | **0.62 (0.45-0.84)** | **0.002** | 1.43 (0.89-2.31) | 0.144 | 1.43 (0.72-2.81) | 0.305 |
| Typical angina | **1.42 (1.09-1.87)** | **0.011** | 1.00 (0.70-1.43) | 0.988 | **1.98 (1.21-3.25)** | **0.007** | **2.70 (1.37-5.32)** | **0.004** |

Displayed are hazard ratios (HR) with 95% confidence intervals (CI) from univariable Cox proportional hazards models. CAD = coronary artery disease, MI = myocardial infarction, uAP = unstable angina pectoris.

**Table S3. Full-Analysis Set**

| **All patients**  **(N=2271)** | **Crude hazard ratios** | | | | **Adjusted hazard ratios** | | | |
| --- | --- | --- | --- | --- | --- | --- | --- | --- |
|  | **Abnormal**  **AI-QCT_ischemia_ result**  **(N=900)** | **Normal**  **AI-QCT_ischemia_ result**  **(N=1371)** | **HR (95% CI)** | **p-value** | **N**  **Patients** | **N**  **Events** | **HR (95% CI)**  **adjusted** | **p-value**  **adjusted** |
| **Death, MI or uAP, n (%)** | 159 (17.7%) | 96 (7.0%) | 2.47 (1.91-3.18) | <0.001 | 2271 | 255 | 1.81 (1.40-2.36) | <0.001^1^ |
| **Death, n (%)** | 68 (12.4%) | 105 (6.1%) | 1.89 (1.40-2.56) | <0.001 | 2271 | 173 | 1.37 (1.01-1.87) | 0.044^2^ |
| **MI, n (%)** | 51 (5.7%) | 17 (1.2%) | 4.45 (2.57-7.70) | <0.001 | 2271 | 68 | 3.46 (1.98-6.05) | <0.001^3^ |
| **uAP, n (%)** | 27 (3.0%) | 7 (0.5%) | 5.71 (2.49-13.13) | <0.001 | 2271 | 34 | 4.48 (1.93-10.41) | <0.001^4^ |

Displayed are numbers (percentage) of first events and hazard ratios (HR) with 95% confidence intervals (CI) from Cox proportional hazards models. Patients with unevaluable AI-QCT_ischemia_ (n=391) were classified as having an abnormal AI-QCT_ischemia_ result. HRs were adjusted for covariates with significant univariable associations with the reported endpoints (**Table S2**): ^1^age, sex, hypertension, diabetes, smoking, typical angina; ^2^age, hypertension, diabetes, smoking, family history of coronary artery diease; ^3^age, hypertension, smoking, dyslipidemia, typical angina; ^4^age, hypertension, typical angina. AI-QCT = artificial intelligence-guided quantitative computed tomography, MI = myocardial infarction, uAP = unstable angina pectoris.

**Table S4. Multivariable Cox Models and C-Indexes Primary Endpoint for Women**

| **Death, MI, uAP**  **1046 Patients**  **94 Events** | **Clinical model (1)** | | **AI-QCT_ischemia_ model (2)** | |
| --- | --- | --- | --- | --- |
|  | **HR (95% CI)** | **p-value** | **HR (95% CI)** | **p-value** |
| AI-QCT_ischemia_ | - | - | **2.03 (1.31-3.13)** | **0.001** |
| Age, per 1 year | **1.10 (1.07-1.13)** | **<0.001** | **1.09 (1.06-1.12)** | **<0.001** |
| Hypertension | 1.14 (0.73-1.79) | 0.556 | 1.07 (0.68-1.67) | 0.781 |
| Diabetes mellitus | 1.06 (0.61-1.84) | 0.837 | 1.00 (0.58-1.75) | 0.991 |
| Smoker | 1.57 (0.99-2.48) | 0.055 | 1.36 (0.85-2.18) | 0.193 |
| Typical angina | 1.25 (0.80-1.96) | 0.331 | 1.20 (0.76-1.88) | 0.432 |
| **C-index**  **(95% CI)** | **0.673**  **(0.621-0.724)** | **<0.001** | **0.694**  **(0.644-0.743)** | **<0.001** |
| **C-index difference**  **(95% CI)** | **Model 2-Model 1** | | **p-value** | |
|  | 0.021 | | 0.070 | |

Displayed are hazard ratios (HR) with 95% confidence intervals (CI) from multivariable Cox proportional hazards models, C-index per model, and difference in C-indexes between the models. AI-QCT = artificial intelligence-guided quantitative computed tomography, MI = myocardial infarction, uAP = unstable angina pectoris.

**Table S5. Multivariable Cox Models and C-Indexes Primary Endpoint for Men**

| **Death, MI, uAP**  **834 Patients**  **112 Events** | **Clinical model (1)** | | **AI-QCT_ischemia_ model (2)** | |
| --- | --- | --- | --- | --- |
|  | **HR (95% CI)** | **p-value** | **HR (95% CI)** | **p-value** |
| AI-QCT_ischemia_ | - | - | **1.92 (1.28-2.87)** | **0.001** |
| Age, per 1 year | **1.06 (1.03-1.08)** | **<0.001** | **1.04 (1.01-1.07)** | **0.004** |
| Hypertension | 1.44 (0.95-1.08) | 0.083 | 1.38 (0.92-2.08) | 0.118 |
| Diabetes mellitus | **1.55 (1.01-2.38)** | **0.044** | 1.47 (0.96-2.25) | 0.079 |
| Smoker | **1.81 (1.24-2.65)** | **0.002** | **1.82 (1.24-2.65)** | **0.002** |
| Typical angina | **1.62 (1.10-2.41)** | **0.016** | 1.44 (0.97-2.15) | 0.071 |
| **C-index**  **(95% CI)** | **0.702**  **(0.654-0.749)** | **<0.001** | **0.720**  **(0.674-0.766)** | **<0.001** |
| **C-index difference**  **(95% CI)** | **Model 2-Model 1** | | **p-value** | |
|  | 0.018  (-0.006 to 0.043) | | 0.141 | |

Displayed are hazard ratios (HR) with 95% confidence intervals (CI) from multivariable Cox proportional hazards models, C-index per model, and difference in C-indexes between the models. AI-QCT = artificial intelligence-guided quantitative computed tomography, MI = myocardial infarction, uAP = unstable angina pectoris.

**Table S6. Baseline Characteristics of Patients with ≤50% Visual Diameter Stenosis**

|  | **N** | **Abnormal**  **AI-QCT_ischemia_ result**  **(N=113)** | **N** | **Normal**  **AI-QCT_ischemia_ result**  **(N=1260)** | **p-value** |
| --- | --- | --- | --- | --- | --- |
| Age, years | 113 | 67 [62-72] | 1260 | 62 [55-68] | <0.001 |
| Sex (female), n (%) | 113 | 56 (49.6%) | 1260 | 789 (62.6%) | 0.006 |
| Hypertension, n (%) | 113 | 79 (69.9%) | 1260 | 632 (50.2%) | <0.001 |
| Dyslipidemia, n (%) | 113 | 74 (65.5%) | 1260 | 770 (61.1%) | 0.360 |
| Current smoker, n (%) | 113 | 16 (14.2%) | 1260 | 149 (11.8%) | 0.465 |
| Previous smoker, n (%) | 113 | 28 (24.8%) | 1260 | 227 (18.0%) | 0.077 |
| BMI, kg/m2 | 89 | 27.2  [24.2-29.0] | 682 | 27.2  [24.4-30.9] | 0.267 |
| Diabetes mellitus, n (%) | 113 | 21 (18.6%) | 1260 | 152 (12.1%) | 0.045 |
| Prediabetes^*^, n (%) | 113 | 14 (12.4%) | 1260 | 161 (12.8%) | 0.906 |
| Family history of CAD, n (%) | 113 | 51 (45.1%) | 1260 | 610 (48.4%) | 0.504 |
| Typical AP, n (%) | 113 | 30 (26.6%) | 1260 | 254 (20.2%) | 0.108 |
| NYHA class  I  II  III | 70 | 41 (58.6%)  25 (35.7%)  4 (5.7%) | 774 | 512 (66.1%)  245 (31.7%)  17 (2.2%) | 0.130 |
| Visual diameter stenosis, n (%)  0%  1-50%  >50% | 113 | 1 (0.9%)  112 (99.1%)  0 (0.0%) | 1260 | 542 (43.0%)  718 (57.0%)  0 (0.0%) | <0.001 |
| Agatston Coronary Calcium Score | 92 | 328  [201-552] | 1046 | 2  [0-64] | <0.001 |
| Downstream PET performed, n (%) | 113 | 76 (67.3) | 1260 | 163 (12.9%) | <0.001 |
| Elective referral for ICA (within 6 months), n (%) | 113 | 19 (16.8%) | 1260 | 109 (8.7%) | 0.004 |
| Early revascularization (within 6 months, PCI or CABG), n (%) | 113 | 6 (5.3%) | 1260 | 4 (0.3%) | <0.001 |
| Early PCI (within 6 months), n (%) | 113 | 6 (5.3%) | 1260 | 4 (0.3%) | <0.001 |

| Early CABG (within 6 months), n (%) | 113 | 0 (0.0%) | 1260 | 0 (0.0%) | - |
| --- | --- | --- | --- | --- | --- |
| Antiplatelet drug  (Aspirin or other), n (%) | 113 | 49 (43.4%) | 1260 | 503 (39.9%) | 0.475 |
| Anticoagulation, n (%) | 113 | 12 (10.6%) | 1260 | 81 (6.4%) | 0.089 |
| Lipid-lowering drug, n (%) | 113 | 55 (48.7%) | 1260 | 446 (35.4%) | 0.005 |
| Betablocker, n (%) | 113 | 47 (41.6%) | 1260 | 500 (39.7%) | 0.691 |
| Long-acting nitrate, n (%) | 113 | 9 (8.0%) | 1260 | 84 (6.7%) | 0.599 |
| Calcium channel blocker, n (%) | 113 | 26 (23.0%) | 1260 | 145 (11.5%) | <0.001 |
| ACE inhibitor, n (%) | 113 | 23 (20.4%) | 1260 | 182 (14.4%) | 0.091 |
| AT II antagonist, n (%) | 113 | 21 (18.6%) | 1260 | 231 (18.3%) | 0.947 |
| Diuretic, n (%) | 113 | 31 (27.4%) | 1260 | 193 (15.3%) | 0.001 |
| Antiarrhythmic drug, n (%) | 113 | 2 (1.8%) | 1260 | 30 (2.4%) | 0.680 |

Values are n (%) or mean (±standard deviation (SD) or median [interquartile range (IQR)]. P-values are from Mann Withney U tests or Chi-square tests. ^*^Prediabetes was defined as HbA1c 6.0-6.5%, or fasting glucose 6.1-6.9 mmol/l or impaired glucose tolerance (2h plasma glucose 7.8-11.0 mmol/l in a 75 oral glucose tolerance test). AI-QCT = artificial intelligence-guided quantitative computed tomography, ACE = angiotensin converting enzyme, AP = angina pectoris, AT II = angiotensin II, BMI = body mass index, CABG = coronary artery bypass grafting, CAD = coronary artery disease, FFR = fractional flow reserve, ICA = invasive coronary angiography, PCI = percutaneous coronary intervention, PET = positron emission tomography.

**Table S7. Baseline Characteristics of Patients with >50% Visual Diameter Stenosis**

|  | **N** | **Abnormal**  **AI-QCT_ischemia_ result**  **(N=378)** | **N** | **Normal**  **AI-QCT_ischemia_ result**  **(N=96)** | **p-value** |
| --- | --- | --- | --- | --- | --- |
| Age, years | 378 | 66 [60-71] | 96 | 67 [58-71] | 0.359 |
| Sex (female), n (%) | 378 | 130 (34.4%) | 96 | 47 (49.0%) | 0.008 |
| Hypertension, n (%) | 378 | 262 (69.3%) | 96 | 64 (66.7%) | 0.617 |
| Dyslipidemia, n (%) | 378 | 284 (75.1%) | 96 | 66 (68.8%) | 0.204 |
| Current smoker, n (%) | 378 | 56 (14.8%) | 96 | 15 (15.6%) | 0.843 |
| Previous smoker, n (%) | 378 | 107 (28.3%) | 96 | 25 (26.0%) | 0.658 |
| BMI, kg/m2 | 330 | 27.7  [24.9-31.2] | 81 | 28.7  [25.4-31.8] | 0.220 |
| Diabetes mellitus, n (%) | 378 | 86 (22.8%) | 96 | 25 (26.0%) | 0.497 |
| Prediabetes^*^, n (%) | 378 | 77 (20.4%) | 96 | 14 (14.6%) | 0.199 |
| Family history of CAD, n (%) | 378 | 167 (44.2%) | 96 | 41 (42.7%) | 0.795 |
| Typical AP, n (%) | 378 | 125 (33.1%) | 96 | 27 (28.1%) | 0.354 |
| NYHA class  I  II  III | 240 | 108 (45.0%)  112 (46.7%)  20 (8.3%) | 59 | 31 (52.5%)  25 (42.4%)  3 (5.1%) | 0.492 |
| Visual diameter stenosis, n (%)  0%  1-50%  >50% | 378 | 0 (0.0%)  0 (0.0%)  378 (100.0%) | 96 | 0 (0.0%)  0 (0.0%)  96 (100.0%) | - |
| Agatston Coronary Calcium Score | 310 | 602  [260-1355] | 76 | 192  [60-375] | <0.001 |
| Downstream PET performed, n (%) | 378 | 323 (85.5%) | 96 | 71 (74.0%) | 0.007 |
| Elective referral for ICA (within 6 months), n (%) | 378 | 243 (64.3%) | 96 | 35 (36.5%) | <0.001 |
| Early revascularization (within 6 months, PCI or CABG), n (%) | 378 | 172 (54.5%) | 96 | 16 (16.7%) | <0.001 |
| Early PCI (within 6 months), n (%) | 378 | 139 (36.8%) | 96 | 15 (15.6%) | <0.001 |

| Early CABG (within 6 months), n (%) | 378 | 37 (9.8%) | 96 | 1 (1.0%) | 0.005 |
| --- | --- | --- | --- | --- | --- |
| Antiplatelet drug  (Aspirin or other), n (%) | 378 | 227 (60.0%) | 96 | 48 (50.0%) | 0.075 |
| Anticoagulation, n (%) | 378 | 30 (7.9%) | 96 | 7 (7.3%) | 0.833 |
| Lipid-lowering drug, n (%) | 378 | 212 (56.1%) | 96 | 52 (54.2%) | 0.735 |
| Betablocker, n (%) | 378 | 214 (56.6%) | 96 | 49 (51.0%) | 0.327 |
| Long-acting nitrate, n (%) | 378 | 44 (11.6%) | 96 | 14 (14.6%) | 0.432 |
| Calcium channel blocker, n (%) | 378 | 77 (20.4%) | 96 | 15 (15.6%) | 0.294 |
| ACE inhibitor, n (%) | 378 | 89 (23.5%) | 96 | 21 (21.9%) | 0.729 |
| AT II antagonist, n (%) | 378 | 96 (25.4%) | 96 | 29 (30.2%) | 0.339 |
| Diuretic, n (%) | 378 | 84 (22.2%) | 96 | 19 (19.8%) | 0.606 |
| Antiarrhythmic drug, n (%) | 378 | 9 (2.4%) | 96 | 3 (3.1%) | 0.679 |

Values are n (%) or mean (±standard deviation (SD) or median [interquartile range (IQR)]. P-values are from Mann Withney U tests or Chi-square tests. ^*^Prediabetes was defined as HbA1c 6.0-6.5%, or fasting glucose 6.1-6.9 mmol/l or impaired glucose tolerance (2h plasma glucose 7.8-11.0 mmol/l in a 75 oral glucose tolerance test). AI-QCT = artificial intelligence-guided quantitative computed tomography, ACE = angiotensin converting enzyme, AP = angina pectoris, AT II = angiotensin II, BMI = body mass index, CABG = coronary artery bypass grafting, CAD = coronary artery disease, FFR = fractional flow reserve, ICA = invasive coronary angiography, PCI = percutaneous coronary intervention, PET = positron emission tomography.

**Table S8. Univariable Cox Regressions for Patients with ≤50% Visual Diameter Stenosis**

| **N=1373** | **Death, MI, or uAP**  **99 events** | | **Death**  **73 events** | | **MI**  **19 events** | | **uAP**  **9 events** | |
| --- | --- | --- | --- | --- | --- | --- | --- | --- |
|  | **HR (95% CI)** | **p-value** | **HR (95% CI)** | **p-value** | **HR (95% CI)** | **p-value** | **HR (95% CI)** | **p-value** |
| Age, per 1 year | **1.08 (1.06-1.11)** | **<0.001** | **1.09 (1.06-1.12)** | **<0.001** | 1.05 (0.98-1.09) | 0.175 | **1.10 (1.01-1.19)** | **0.021** |
| Sex  (male vs. female) | 1.31 (0.88-1.94) | 0.187 | 1.28 (0.81-2.03) | 0.295 | 1.13 (0.46-2.82) | 0.787 | 1.26 (0.34-4.71) | 0.727 |
| Hypertension | **1.59 (1.06-2.39)** | **0.024** | 1.41 (0.88-2.24) | 0.151 | 2.16 (0.82-5.68) | 0.119 | 3.43 (0.71-16.51) | 0.125 |
| Diabetes mellitus | 1.17 (0.65-2.10) | 0.598 | 1.70 (0.93-3.09) | 0.085 | - | - | - | - |
| Smoker | **1.60 (1.07-2.39)** | **0.021** | 1.50 (0.94-2.40) | 0.086 | 1.95 (0.79-4.79) | 0.148 | 1.75 (0.47-6.51) | 0.405 |
| Dyslipidemia | 0.77 (0.52-1.15) | 0.208 | 0.81 (0.51-1.29) | 0.381 | 1.01 (0.40-2.57) | 0.985 | 0.49 (0.13-1.83) | 0.291 |
| Family history  of CAD | 0.84 (0.57-1.25) | 0.400 | **0.58 (0.36-0.94)** | **0.026** | 1.79 (0.70-4.54) | 0.222 | 2.08 (0.52-8.31) | 0.301 |
| Typical angina | **1.70 (1.10-2.63)** | **0.016** | 1.24 (0.72-2.13) | 0.446 | **2.93 (1.17-7.34)** | **0.022** | 3.16 (0.85-11.77) | 0.087 |

Displayed are hazard ratios (HR) with 95% confidence intervals (CI) from univariable Cox proportional hazards models. CAD = coronary artery disease, MI = myocardial infarction, uAP = unstable angina pectoris.

**Table S9. Univariable Cox Regressions for Patients with >50% Visual Diameter Stenosis**

| **N=474** | **Death, MI, or uAP**  **107 events** | | **Death**  **62 events** | | **MI**  **40 events** | | **uAP**  **22 events** | |
| --- | --- | --- | --- | --- | --- | --- | --- | --- |
|  | **HR (95% CI)** | **p-value** | **HR (95% CI)** | **p-value** | **HR (95% CI)** | **p-value** | **HR (95% CI)** | **p-value** |
| Age, per 1 year | **1.04 (1.01-1.06)** | **0.003** | **1.06 (1.02-1.09)** | **0.001** | 1.04 (1.00-1.08) | 0.059 | 1.01 (0.96-1.06) | 0.696 |
| Sex  (male vs. female) | 0.94 (0.64-1.40) | 0.777 | 0.86 (0.51-1.44) | 0.573 | 0.97 (0.51-1.84) | 0.918 | 0.83 (0.35-1.94) | 0.662 |
| Hypertension | 1.33 (0.87-2.05) | 0.191 | 1.26 (0.72-2.20) | 0.421 | 1.42 (0.69-2.90) | 0.339 | 2.09 (0.71-6.17) | 0.183 |
| Diabetes mellitus | 1.51 (1.00-2.28) | 0.050 | **1.88 (1.12-3.17)** | **0.017** | 1.64 (0.85-3.18) | 0.143 | 0.32 (0.08-1.38) | 0.127 |
| Smoker | 1.27 (0.87-1.85) | 0.224 | 1.42 (0.87-2.34) | 0.165 | 1.47 (0.79-2.73) | 0.225 | 0.97 (0.42-2.27) | 0.947 |
| Dyslipidemia | 0.88 (0.57-1.34) | 0.543 | 0.63 (0.37-1.07) | 0.088 | 1.98 (0.83-4.72) | 0.123 | 1.18 (0.43-3.19) | 0.751 |
| Family history  of CAD | 0.90 (0.61-1.32) | 0.577 | 0.75 (0.45-1.25) | 0.270 | 1.15 (0.62-2.13) | 0.668 | 1.29 (0.56-2.98) | 0.549 |
| Typical angina | 1.13 (0.76-1.69) | 0.555 | 0.72 (0.40-1.27) | 0.250 | 1.44 (0.76-2.71) | 0.261 | 1.82 (0.79-4.22) | 0.161 |

Displayed are hazard ratios (HR) with 95% confidence intervals (CI) from univariable Cox proportional hazards models. CAD = coronary artery disease, MI = myocardial infarction, uAP = unstable angina pectoris.

.

**Table S10. Multivariable Cox Models and C-Indexes Primary Endpoint for Patients**

**with Visual No/Non-Obstructive Stenosis (≤50%)**

| **Death, MI, uAP**  **1373 Patients**  **99 Events** | **Clinical model (1)** | | **Clinical+AI-QCT_ischemia_ model (2)** | |
| --- | --- | --- | --- | --- |
|  | **HR (95% CI)** | **p-value** | **HR (95% CI)** | **p-value** |
| AI-QCT_ischemia_ | - | - | **1.74 (1.04-2.90)** | **0.035** |
| Age, per 1 year | **1.09 (1.06-1.12)** | **<0.001** | **1.08 (1.06-1.11)** | **<0.001** |
| Sex  (male vs. female) | 1.44 (0.95-2.18) | 0.085 | 1.38 (0.91-2.09) | 0.128 |
| Hypertension | 1.21 (0.80-1.84) | 0.371 | 1.19 (0.78-1.82) | 0.411 |
| Diabetes mellitus | 1.04 (0.57-1.90) | 0.888 | 1.00 (0.55-1.82) | 0.995 |
| Smoker | **1.92 (1.26-2.94)** | **0.003** | **1.85 (1.21-2.82)** | **0.005** |
| Typical angina | **1.59 (1.03-2.46)** | **0.036** | 1.53 (0.99-2.38) | 0.055 |
| **C-index**  **(95% CI)** | **0.722**  **(0.670-0.774)** | **<0.001** | **0.726**  **(0.674-0.777)** | **<0.001** |
| **C-index difference**  **(95% CI)** | **Model 2-Model 1** | | **p-value** | |
|  | 0.004  (-0.009 to 0.017) | | 0.535 | |

Displayed are hazard ratios (HR) with 95% confidence intervals (CI) from multivariable Cox regressions, C-index per model, and difference in C-indexes between the models. AI-QCT = artificial intelligence-guided quantitative computed tomography, MI = myocardial infarction, uAP = unstable angina pectoris.

**Table S11. Multivariable Cox Models and C-Indexes Primary Endpoint for Patients**

**with Visual Obstructive Stenosis (>50%)**

| **Death, MI, uAP**  **474 Patients**  **107 Events** | **Clinical model (1)** | | **Clinical+AI-QCT_ischemia_ model (2)** | |
| --- | --- | --- | --- | --- |
|  | **HR (95% CI)** | **p-value** | **HR (95% CI)** | **p-value** |
| AI-QCT_ischemia_ | - | - | 1.27 (0.75-2.14) | 0.374 |
| Age, per 1 year | **1.04 (1.01-1.07)** | **0.003** | **1.04 (1.01-1.07)** | **0.004** |
| Sex  (male vs. female) | 1.07 (0.71-1.61) | 0.747 | 1.05 (0.70-1.58) | 0.814 |
| Hypertension | 1.26 (0.81-1.96) | 0.301 | 1.26 (0.81-1.96) | 0.306 |
| Diabetes mellitus | 1.36 (0.90-2.07) | 0.146 | 1.38 (0.91-2.11) | 0.129 |
| Smoker | 1.35 (0.92-1.9) | 0.129 | 1.35 (0.92-1.99) | 0.128 |
| Typical angina | 1.13 (0.75-1.69) | 0.557 | 1.12 (0.75-1.68) | 0.575 |
| **C-index**  **(95% CI)** | **0.602**  **(0.548-0.656)** | **<0.001** | **0.604**  **(0.550-0.659)** | **<0.001** |
| **C-index difference**  **(95% CI)** | **Difference Model 2-Model 1** | | **p-value** | |
|  | 0.002  (-0.012 to 0.017) | | 0.741 | |

Displayed are hazard ratios (HR) with 95% confidence intervals (CI) from multivariable Cox regressions, C-index per model, and difference in C-indexes between the models. AI-QCT = artificial intelligence-guided quantitative computed tomography, MI = myocardial infarction, uAP = unstable angina pectoris.

**Table S12. Univariable Cox Regressions Primary Endpoint for Patients With and Without Early Revascularization**

| **Patients without revascularization**  **(N=1676)** | **Death, MI, or uAP**  **164 events** | | **Patients with revascularization**  **(N=204)** | **Death, MI, or uAP**  **42 events** | |
| --- | --- | --- | --- | --- | --- |
|  | **HR (95% CI)** | **p-value** |  | **HR (95% CI)** | **p-value** |
| Age, per 1 year | **1.07 (1.05-1.09)** | **<0.001** | Age, per 1 year | **1.05 (1.00-1.09)** | **0.028** |
| Sex  (male vs. female) | **1.52 (1.12-2.07)** | **0.007** | Sex  (male vs. female) | 0.64 (0.34-1.20) | 0.166 |
| Hypertension | **1.77 (1.28-2.45)** | **0.001** | Hypertension | 1.35 (0.70-2.59) | 0.374 |
| Diabetes mellitus | **1.17 (1.14-2.42)** | **0.009** | Diabetes mellitus | 1.41 (0.72-2.75) | 0.321 |
| Smoker | **1.60 (1.07-2.39)** | **0.021** | Smoker | 1.01 (0.55-1.85) | 0.981 |
| Dyslipidemia | 0.89 (0.65-1.21) | 0.450 | Dyslipidemia | 0.85 (0.41-1.79) | 0.671 |
| Family history  of CAD | 0.77 (0.56-1.05) | 0.094 | Family history  of CAD | 1.10 (0.60-2.02) | 0.746 |
| Typical angina | **1.46 (1.03-2.06)** | **0.033** | Typical angina | 1.38 (0.75-2.52) | 0.300 |

Displayed are hazard ratios (HR) with 95% confidence intervals (CI) from univariable Cox proportional hazards models. CAD = coronary artery disease, MI = myocardial infarction, uAP = unstable angina pectoris.
